# Fipronil and permethrin combination: a novel ectoparasiticide for dogs

**DOI:** 10.1186/s13071-015-0672-1

**Published:** 2015-01-27

**Authors:** Luís Cardoso

**Affiliations:** Department of Veterinary Sciences, School of Agrarian and Veterinary Sciences, University of Trás-os-Montes e Alto Douro (UTAD), Vila Real, Portugal

Arthropods may be ectoparasites of dogs, representing a nuisance and causing direct damage such as blood loss, irritation, skin lesions, hypersensitivity reactions, toxicosis and even paralysis. Many of those invertebrates are also a potential indirect threat to their vertebrate hosts due to the transmission, mainly while feeding on blood, of a range of vector-borne pathogens, including bacteria, protozoa, helminths and viruses, many of which are of zoonotic concern. Ticks, together with fleas, mosquitoes, sand flies and other insects, are among the haematophagous ectoparasites that have a major impact on the well-being and health not only of dogs but also of humans, thus providing factual examples of the “One Health” concept. The vector-borne diseases of dogs include anaplasmosis, babesiosis, bartonellosis, borreliosis, dipylidiosis, dirofilariosis, ehrlichiosis, leishmaniosis and rickettsiosis, most of which represent emergent veterinary medical and public health problems in many geographical regions worldwide.

Prevention of canine vector-borne diseases largely relies on ectoparasite control, aiming at protecting dogs and simultaneously limiting the risk of zoonotic infections. Due to changes in the distribution of arthropods, along with those in the lifestyle of pets, it is advisable to maintain protection against ectoparasites year-round. Regular application of effective anti-vector products on dogs remains the best approach to control infestations and associated diseases. The range of ectoparasiticides has expanded in recent years with the development of new active ingredients and formulations. Many active ingredients are available on the market as insecticides/acaricides, supplying veterinarians and dog owners with a wide variety of products to choose from, such as sprays, spot-on pipettes, impregnated collars and orally administered preparations. One current approach in the development of tools for parasite control is the use of existing molecules in combinations.

This *Parasites & Vectors* article collection represents a series of seven papers describing studies conducted on the efficacy of a novel combination of fipronil and permethrin in a spot-on formulation for dogs (Frontline Tri-Act®/Frontect®, Merial). Fipronil is a phenylpyrazole with both insecticidal and acaricidal properties; permethrin is a pyrethroid which combines insecticidal, acaricidal and repellent activities; both diffuse across the skin and accumulate in sebaceous glands and cutaneous lipids, remaining active for weeks. Although there is substantial available scientific literature on the efficacy of fipronil and permethrin as separate molecules, no previous study has been published on the performance of their combination. The efficacy of the current formulation has been demonstrated in controlled studies in dogs.

In five different studies with adult *Ctenocephalides felis*, a single application of fipronil and permethrin provided excellent levels of efficacy against fleas on dogs at 24 and 48 hours post-treatment or post-infestation, during one month. In two of those studies, efficacy was maintained at 100% during a full month after treatment in dogs that underwent either three water immersions or one shampooing, simulating an outdoor lifestyle. One of the articles presents interesting results on the repellency of the product against *Ctenocephalides canis* fleas at one hour post-infestation, which is a less investigated parameter of insecticide preparations. A successful flea control program aims at eliminating fleas quickly after infestation and preventing re-infestation from the environment. For fleas, insecticides should have rapid killing or repellent activity. Adult fleas infesting the host must be eliminated, and further infestations by latent environmental stages should be prevented.

The new ectoparasiticide had the ability of killing ticks when administered to infested dogs (immediate or curative efficacy) and to prevent tick re-infestations for at least four weeks after treatment (persistent or protective efficacy). A single topical treatment with the fipronil and permethrin combination provided excellent acaricidal efficacy against both *Ixodes ricinus* and *Rhipicephalus sanguineus*. Regarding *Dermacentor reticulatus*, the new product demonstrated high repellency and prevention of attachment, at 12 and 24 hours after re-infestation, and excellent acaricidal efficacy, within 48 hours, with the effects persisting up to four weeks after treatment. Transmission of most pathogens from tick species to dogs usually requires an initial pre-activation period of about 24 to 48 hours after attachment. Taking these times into account, the results suggest that the application to dogs of the new combination can significantly reduce the potential for transmission of *Babesia canis* as well as other agents of tick-borne diseases.

The new fipronil and permethrin combination demonstrated a high repellency (*i.e.*, an anti-feeding effect) against the bites of *Phlebotomus perniciosus* sand flies, which lasted for three to four weeks. A single topical administration of the product also provided high repellency against mosquitoes (*Aedes aegypti*, *Aedes albopictus* and *Culex pipiens*) on dogs for at least four weeks. High protection (repellency and insecticidal efficacy) of the combination of fipronil and permethrin was also found against the biting stable fly *Stomoxys calcitrans* on dogs for at least five weeks after a single treatment. This overall repellent efficacy by inhibition of blood-feeding may contribute to the protection of dogs from sequelae of bites by mosquitoes and biting flies, as well as from major pathogens transmitted by mosquitoes and sand flies, *i.e.*, *Dirofilaria* spp. and *Leishmania infantum*, respectively.

In conclusion, a long lasting repellent effect against mosquitoes, sand flies and stable flies has been demonstrated, along with a long lasting efficacy against fleas and the main tick species infesting dogs in Europe. For one month following a single topical application, the new fipronil and permethrin ectoparasiticide combination offers a broad spectrum protection against the major canine ectoparasites, protecting the dogs themselves and also minimizing the risk of zoonotic pathogen transmission.

